# Mg_6.75_(OH)_3_(H_0.166_AsO_4_)_3_(HAsO_4_), a member of the *M*_1-_ *_x_M*′_6_(OH)_3_(H_2*x*/3_AsO_4_)_3_(HAsO_4_) family (*M*,*M*′ = Co; Ni)

**DOI:** 10.1107/S1600536813010714

**Published:** 2013-04-24

**Authors:** Matthias Weil

**Affiliations:** a Institute for Chemical Technologies and Analytics, Division of Structural Chemistry, Vienna University of Technology, Getreidemarkt 9/164-SC, A-1060 Vienna, Austria

## Abstract

In the structure of the title compound, magnesium hydroxide hydrogenarsenate (6.75/3/4), two different Mg^2+^ ions, one located on a site with symmetry 3*m*. (occupancy 3/4) and one on a general position, as well as two different AsO_3_(OH) tetra­hedra (symmetry .*m*. with partial occupancy for the H atom for one, and symmetry 3*m*. with full occupancy for the H atom for the other) and one OH^−^ ion (site symmetry .*m*.) are present. Both Mg^2+^ ions are octa­hedrally surrounded by O atoms. The MgO_6_ octa­hedra belonging to the partially occupied Mg^2+^ sites share faces, forming chains along [001]. The other type of MgO_6_ octa­hedra share corners and faces under formation of strands parallel to [001] whereby individual strands are linked through common corner atoms. The two types of AsO_3_(OH) tetra­hedra inter­link the strands and the chains, building up a three-dimensional framework resembling that of the mineral dumortierite. The OH groups were assigned on basis of bond-valence calculations and crystal chemical considerations.

## Related literature

For the isotypic Co and Ni members of the *M*_1-_ *_x_M*′_6_(OH)_3_(H_2*x*/3_AsO_4_)_3_(HAsO_4_) series, see: Hughes *et al.* (2003 ▶). For other reaction products obtained under the given or similar hydro­thermal conditions, see: Weil (2004*a* ▶,*b* ▶). For the crystal structure of dumortierite, see: Alexander *et al.* (1986 ▶). The bond-valence method has been described by Brown (2002 ▶).

## Experimental

### 

#### Crystal data

Mg_6.75_(OH)_3_(H_0.166_AsO_4_)_3_(HAsO_4_)
*M* *_r_* = 772.31
Hexagonal, 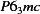

*a* = 12.7651 (3) Å
*c* = 5.0844 (1) Å
*V* = 717.49 (3) Å^3^
*Z* = 2
Mo *K*α radiation
μ = 9.65 mm^−1^
*T* = 296 K
0.30 × 0.02 × 0.02 mm

#### Data collection

Bruker APEXII CCD diffractometer
Absorption correction: multi-scan (*SADABS*; Bruker, 2008 ▶) *T*_min_ = 0.160, *T*_max_ = 0.830
21473 measured reflections
1144 independent reflections
1066 reflections with *I* > 2σ(*I*)
*R*_int_ = 0.051

#### Refinement

*R*[*F* ^2^ > 2σ(*F* ^2^)] = 0.029
*wR*(*F* ^2^) = 0.058
*S* = 1.21
1144 reflections
60 parameters
1 restraint
H-atom parameters not refined
Δρ_max_ = 1.31 e Å^−3^
Δρ_min_ = −2.16 e Å^−3^
Absolute structure: Flack (1983 ▶), 446 Friedel pairs
Flack parameter: 0.022 (16)

### 

Data collection: *APEX2* (Bruker, 2008 ▶); cell refinement: *SAINT* (Bruker, 2008 ▶); data reduction: *SAINT*; program(s) used to solve structure: *SHELXS97* (Sheldrick, 2008 ▶); program(s) used to refine structure: *SHELXL97* (Sheldrick, 2008 ▶); molecular graphics: *ATOMS for Windows* (Dowty, 2006 ▶); software used to prepare material for publication: *publCIF* (Westrip, 2010 ▶).

## Supplementary Material

Crystal structure: contains datablock(s) I, global. DOI: 10.1107/S1600536813010714/vn2069sup1.cif

Structure factors: contains datablock(s) I. DOI: 10.1107/S1600536813010714/vn2069Isup2.hkl

Additional supplementary materials:  crystallographic information; 3D view; checkCIF report

## Figures and Tables

**Table 1:** Selected bond lengths (Å)

Mg1—O1^i^	2.100 (5)
Mg1—O1^ii^	2.102 (5)
Mg1—Mg1^iii^	2.5422 (1)
Mg2—O2	2.018 (3)
Mg2—O2^iv^	2.029 (3)
Mg2—O5	2.036 (3)
Mg2—O3^v^	2.053 (2)
Mg2—O4	2.139 (3)
Mg2—O4^ii^	2.225 (3)
As1—O2	1.678 (2)
As1—O1	1.687 (4)
As1—O3	1.696 (3)
As2—O5	1.644 (3)
As2—O6^ii^	1.710 (8)

Symmetry codes: (i) 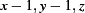; (ii) 
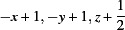
; (iii) 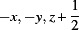; (iv) 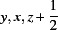; (v) 
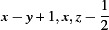
.

**Fig. 1.**
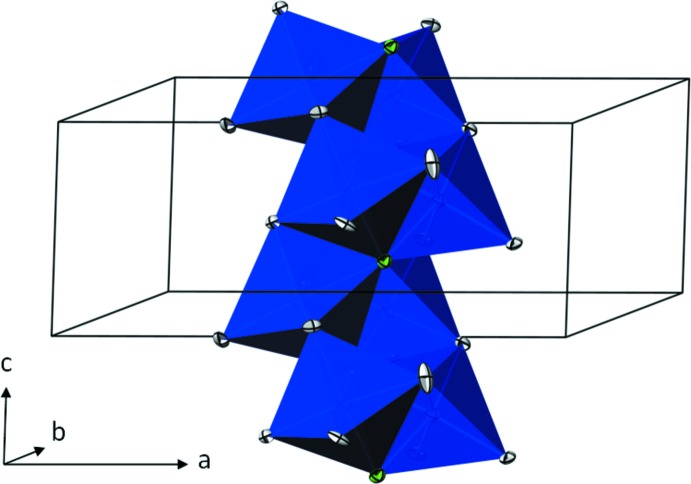
Strands of face- and edge-sharing Mg2O6 octahedra extending parallel to [001]. Atoms are displayed with anisotropic displacement parameters at the 74% level. The O atom (O4) of the OH group is given in green.

**Fig. 2.**
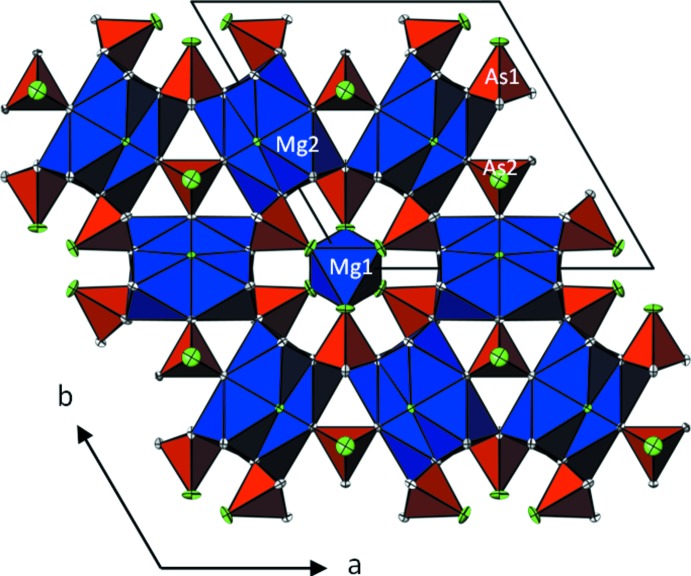
The crystal structure of the title compound in a projection along [001]. MgO6 octahedra are blue, AsO4 tetrahedra red. O atoms are displayed with anisotropic displacement parameters at the 74% level. O atoms belongig to OH groups (with fully or partly occupied H atoms) are given in green.

## supplementary crystallographic information

### Comment

Crystals of the title compound, Mg_6.75_(OH)_3_(H_0.166_AsO_4_)_3_(HAsO_4_), were obtained serendipitously during crystal growth experiments intended to grow a possible descloizite-type phase 'MgHg(AsO_4_)(OH)' under hydrothermal formation conditions, similar to the formation of ZnHg(AsO_4_)(OH) (Weil, 2004*a*).

Mg_6.75_(OH)_3_(H_0.166_AsO_4_)_3_(HAsO_4_) belongs to the isotypic series *M*_1 -_*_x_M*'_6_(OH)_3_(H_2_*_x_*_/3_AsO_4_)_3_(HAsO_4_) for which the Co and Ni members have been structurally determined by Hughes *et al.* (2003). The crystal structures contain two *M*^2+^ ions, one (*M*1) located on a site with symmetry 3*m*. and with varying partial occupancy, and the other (*M*2) located on a general position with full occupancy. Two different AsO_3_(OH) tetrahedra, one (As1) with symmetry .*m*. and partial occupancy for its H atom, and the other (As2) with symmetry 3*m*. and full occupancy for the H atom, as well as one OH^-^ ion (site symmetry .*m*.; full occupancy for the H atom) are also present.

The two metal ions are octahedrally surrounded by O atoms, with Mg—O distances in the range 2.018 (3) to 2.225 (3) Å. The Mg1O_6_ octahedra share faces forming chains parallel to [001]. Mg2O_6_ octahedra share faces and edges forming strands parallel to [001] (Fig. 1). Individual strands are linked with neighbouring strands through common corner atoms. The AsO_3_(OH) tetrahedra flank the chains and strands and link both motifs into a three-dimensional framework (Fig. 2).

Since the protons required for charge balance could not be located from difference maps, the assignment of OH groups was made both from crystal chemical considerations and calculation of bond valence sums (Brown, 2002). The valence sums for Mg1 (1.99 v.u.), Mg2 (2.13), As1 (5.00) and As2 (5.34) are near the expected values of 2 and 5, respectively. The values of 1.90 for O1, 2.09 for O2, 2.00 for O3, 1.07 for O4, 2.19 for O5 and 1.17 for O6 suggest that O1, O4 and O6 belong to hydroxide groups; the occupancy of the attached H atom sites of O1 is 0.166 and is dependent on the occupancy of the Mg1 site to which the As1O_3_(OH) group is attached. H atoms attached to O4 and O6 are fully occupied. O4 is the OH group bonded to four Mg2^2+^ cations. For this bridging *µ*_4_ group the longest Mg—O distances of 2.139 (3) and 2.225 (3) Å are observed. O6 is the OH group of an As2O_3_(OH) tetrahedron; it is solely bonded to As2 and is a much longer (As2—O6(H) = 1.710 (8) Å) than the As2—O5 bonds (1.644 (3) Å) which is typical for AsO_3_(OH) units.

An interesting feature of this structure type is the short *M*···*M* contact within the chains of face-sharing *M*1O_6_ octahedra running along [001]. The observed Mg···Mg distance of 2.5422 (1) Å corresponds to c/2 and lies between the respective distances of 2.5460 (1) Å for the Co and of 2.4843 (5) Å for the Ni member (Hughes *et al.*, 2003).

The topological similarities between the framework structure of the title compound and that of the minerals dumortierite (Alexander *et al.*, 1986) and cancrinite has been discussed in detail by Hughes *et al.* (2003).

### Experimental

200 mg of an amorphous precipitation product obtained by reacting MgCO_3_ and arsenic acid (*ca* 20%_wt_) was mixed with 300 mg HgO and placed in a Teflon container (volume 10 ml) that was filled up with two-thirds of its volume with water. The inlay was placed in a steel autoclave and heated at 493 K for two weeks. Besides colourless needle-shaped crystals of the title compound, recrystallized HgO and α-(Hg_2_)_3_(AsO_4_)_2_ (Weil, 2004*b*) were also present, as determined by single-crystal X-ray diffraction of selected crystals.

### Refinement

The atomic coordinates of the isotypic Co compound (Hughes *et al.*, 2003) were used as starting parameters for refinement. The site occupation factor of Mg1 was refined freely; no significant deviation from full occupancy for Mg2 was observed. Hydrogen atoms could not be located reliably from difference maps and hence were not included in the refinement. According to the occupancy of Mg1, the overall number of H atoms was calculated as 4.5 for charge compensation. The maximum and minimum remaining electron densities were found 1.14 and 1.21 Å away from atom As2.

### Crystal data

**Table:**

Mg_6.75_(OH)_3_(H_0.166_AsO_4_)_3_(HAsO_4_)	*D*_x_ = 3.575 Mg m^−^^3^
*M**_r_* = 772.31	Mo *K*α radiation, λ = 0.71073 Å
Hexagonal, *P*6_3_*m**c*	Cell parameters from 5443 reflections
Hall symbol: P 6c -2c	θ = 6.2–34.4°
*a* = 12.7651 (3) Å	µ = 9.65 mm^−^^1^
*c* = 5.0844 (1) Å	*T* = 296 K
*V* = 717.49 (3) Å^3^	Needle, colourless
*Z* = 2	0.30 × 0.02 × 0.02 mm
*F*(000) = 739	

### Data collection

**Table:**

Bruker APEXII CCD diffractometer	1144 independent reflections
Radiation source: fine-focus sealed tube	1066 reflections with *I* > 2σ(*I*)
Graphite monochromator	*R*_int_ = 0.051
ω and φ scans	θ_max_ = 36.2°, θ_min_ = 1.8°
Absorption correction: multi-scan (*SADABS*; Bruker, 2008)	*h* = −21→21
*T*_min_ = 0.160, *T*_max_ = 0.830	*k* = −21→21
21473 measured reflections	*l* = −5→8

### Refinement

**Table:**

Refinement on *F*^2^	H-atom parameters not refined
Least-squares matrix: full	*w* = 1/[σ^2^(*F*_o_^2^) + (0.0061*P*)^2^ + 2.8884*P*] where *P* = (*F*_o_^2^ + 2*F*_c_^2^)/3
*R*[*F*^2^ > 2σ(*F*^2^)] = 0.029	(Δ/σ)_max_ < 0.001
*wR*(*F*^2^) = 0.058	Δρ_max_ = 1.31 e Å^−^^3^
*S* = 1.21	Δρ_min_ = −2.16 e Å^−^^3^
1144 reflections	Extinction correction: *SHELXL97* (Sheldrick, 2008), Fc^*^=kFc[1+0.001xFc^2^λ^3^/sin(2θ)]^-1/4^
60 parameters	Extinction coefficient: 0.0015 (4)
1 restraint	Absolute structure: Flack (1983), 446 Friedel pairs
Primary atom site location: isomorphous structure methods	Flack parameter: 0.022 (16)

### Special details

**Table:**

Geometry. All e.s.d.'s (except the e.s.d. in the dihedral angle between two l.s. planes) are estimated using the full covariance matrix. The cell e.s.d.'s are taken into account individually in the estimation of e.s.d.'s in distances, angles and torsion angles; correlations between e.s.d.'s in cell parameters are only used when they are defined by crystal symmetry. An approximate (isotropic) treatment of cell e.s.d.'s is used for estimating e.s.d.'s involving l.s. planes.
Refinement. Refinement of *F*^2^ against ALL reflections. The weighted *R*-factor *wR* and goodness of fit *S* are based on *F*^2^, conventional *R*-factors *R* are based on *F*, with *F* set to zero for negative *F*^2^. The threshold expression of *F*^2^ > σ(*F*^2^) is used only for calculating *R*-factors(gt) *etc*. and is not relevant to the choice of reflections for refinement. *R*-factors based on *F*^2^ are statistically about twice as large as those based on *F*, and *R*- factors based on ALL data will be even larger.

### Fractional atomic coordinates and isotropic or equivalent isotropic displacement parameters (Å^2^)

**Table:**

	*x*	*y*	*z*	*U*_iso_*/*U*_eq_	Occ. (<1)
Mg1	0.0000	0.0000	0.8698 (12)	0.0227 (15)	0.750 (14)
Mg2	0.65062 (9)	0.57400 (10)	0.5491 (3)	0.0085 (2)	
As1	0.69764 (4)	0.848821 (19)	0.57265 (9)	0.00778 (9)	
As2	0.6667	0.3333	0.78398 (17)	0.00550 (14)	
O1	0.8487 (3)	0.92434 (17)	0.6201 (9)	0.0257 (10)	
O2	0.6564 (2)	0.7229 (2)	0.3969 (5)	0.0100 (4)	
O3	0.6168 (3)	0.80839 (17)	0.8561 (7)	0.0115 (6)	
O4	0.52447 (15)	0.47553 (15)	0.2427 (7)	0.0091 (6)	
O5	0.59609 (16)	0.40391 (16)	0.6823 (8)	0.0173 (8)	
O6	0.3333	0.6667	0.6202 (15)	0.0319 (19)	

### Atomic displacement parameters (Å^2^)

**Table:**

	*U*^11^	*U*^22^	*U*^33^	*U*^12^	*U*^13^	*U*^23^
Mg1	0.0120 (13)	0.0120 (13)	0.044 (4)	0.0060 (6)	0.000	0.000
Mg2	0.0103 (4)	0.0092 (4)	0.0073 (5)	0.0059 (3)	−0.0005 (4)	0.0001 (4)
As1	0.01061 (18)	0.00811 (12)	0.00544 (16)	0.00530 (9)	−0.0004 (2)	−0.00021 (10)
As2	0.00468 (17)	0.00468 (17)	0.0071 (3)	0.00234 (9)	0.000	0.000
O1	0.0089 (14)	0.0320 (17)	0.029 (3)	0.0044 (7)	−0.0038 (16)	−0.0019 (8)
O2	0.0141 (10)	0.0099 (9)	0.0072 (10)	0.0070 (8)	−0.0016 (8)	−0.0015 (8)
O3	0.0188 (16)	0.0110 (10)	0.0074 (14)	0.0094 (8)	0.0073 (12)	0.0036 (6)
O4	0.0099 (10)	0.0099 (10)	0.0082 (14)	0.0053 (11)	0.0009 (5)	−0.0009 (5)
O5	0.0099 (10)	0.0099 (10)	0.034 (2)	0.0065 (12)	−0.0037 (7)	0.0037 (7)
O6	0.042 (3)	0.042 (3)	0.011 (4)	0.0212 (15)	0.000	0.000

### Geometric parameters (Å, º)

**Table:**

Mg1—O1^i^	2.100 (5)	Mg2—O3^x^	2.053 (2)
Mg1—O1^ii^	2.100 (5)	Mg2—O4	2.139 (3)
Mg1—O1^iii^	2.100 (5)	Mg2—O4^iv^	2.225 (3)
Mg1—O1^iv^	2.102 (5)	As1—O2	1.678 (2)
Mg1—O1^v^	2.102 (5)	As1—O2^xi^	1.678 (2)
Mg1—O1^vi^	2.102 (5)	As1—O1	1.687 (4)
Mg1—Mg1^vii^	2.5422 (1)	As1—O3	1.696 (3)
Mg1—Mg1^viii^	2.5422 (1)	As2—O5^ii^	1.644 (3)
Mg2—O2	2.018 (3)	As2—O5^xii^	1.644 (3)
Mg2—O2^ix^	2.029 (3)	As2—O5	1.644 (3)
Mg2—O5	2.036 (3)	As2—O6^iv^	1.710 (8)
			
O1^i^—Mg1—O1^ii^	87.2 (2)	O2—As1—O2^xi^	106.56 (16)
O1^i^—Mg1—O1^iii^	87.2 (2)	O2—As1—O1	110.19 (12)
O1^ii^—Mg1—O1^iii^	87.2 (2)	O2^xi^—As1—O1	110.19 (12)
O1^i^—Mg1—O1^iv^	179.9 (3)	O2—As1—O3	108.02 (11)
O1^ii^—Mg1—O1^iv^	92.81 (9)	O2^xi^—As1—O3	108.02 (11)
O1^iii^—Mg1—O1^iv^	92.81 (9)	O1—As1—O3	113.6 (2)
O1^i^—Mg1—O1^v^	92.81 (9)	O5^ii^—As2—O5^xii^	110.58 (15)
O1^ii^—Mg1—O1^v^	179.9 (3)	O5^ii^—As2—O5	110.58 (15)
O1^iii^—Mg1—O1^v^	92.81 (9)	O5^xii^—As2—O5	110.58 (15)
O1^iv^—Mg1—O1^v^	87.1 (2)	O5^ii^—As2—O6^iv^	108.33 (15)
O1^i^—Mg1—O1^vi^	92.81 (9)	O5^xii^—As2—O6^iv^	108.33 (15)
O1^ii^—Mg1—O1^vi^	92.81 (9)	O5—As2—O6^iv^	108.33 (15)
O1^iii^—Mg1—O1^vi^	179.9 (3)	As1—O1—Mg1^xiii^	151.0 (3)
O1^iv^—Mg1—O1^vi^	87.1 (2)	As1—O1—Mg1^xiv^	134.5 (3)
O1^v^—Mg1—O1^vi^	87.1 (2)	Mg1^xiii^—O1—Mg1^xiv^	74.46 (12)
O2—Mg2—O2^ix^	93.03 (10)	As1—O2—Mg2	122.99 (14)
O2—Mg2—O5	164.45 (12)	As1—O2—Mg2^xv^	137.98 (14)
O2^ix^—Mg2—O5	95.00 (14)	Mg2—O2—Mg2^xv^	98.03 (11)
O2—Mg2—O3^x^	89.84 (12)	As1—O3—Mg2^ix^	121.83 (9)
O2^ix^—Mg2—O3^x^	98.49 (13)	As1—O3—Mg2^xvi^	121.83 (9)
O5—Mg2—O3^x^	102.09 (14)	Mg2^ix^—O3—Mg2^xvi^	115.94 (18)
O2—Mg2—O4	86.36 (11)	Mg2—O4—Mg2^xvii^	84.17 (14)
O2^ix^—Mg2—O4	162.38 (12)	Mg2—O4—Mg2^xiv^	147.49 (17)
O5—Mg2—O4	81.96 (12)	Mg2^xvii^—O4—Mg2^xiv^	88.83 (7)
O3^x^—Mg2—O4	99.12 (14)	Mg2—O4—Mg2^xv^	88.83 (7)
O2—Mg2—O4^iv^	89.76 (11)	Mg2^xvii^—O4—Mg2^xv^	147.49 (17)
O2^ix^—Mg2—O4^iv^	83.86 (11)	Mg2^xiv^—O4—Mg2^xv^	80.23 (13)
O5—Mg2—O4^iv^	77.91 (13)	As2—O5—Mg2^xvii^	134.44 (9)
O3^x^—Mg2—O4^iv^	177.63 (15)	As2—O5—Mg2	134.44 (9)
O4—Mg2—O4^iv^	78.53 (7)	Mg2^xvii^—O5—Mg2	89.51 (15)

Symmetry codes: (i) *x*−1, *y*−1, *z*; (ii) −*y*+1, *x*−*y*, *z*; (iii) −*x*+*y*, −*x*+1, *z*; (iv) −*x*+1, −*y*+1, *z*+1/2; (v) *y*−1, −*x*+*y*, *z*+1/2; (vi) *x*−*y*, *x*−1, *z*+1/2; (vii) −*x*, −*y*, *z*+1/2; (viii) −*x*, −*y*, *z*−1/2; (ix) *y*, *x*, *z*+1/2; (x) *x*−*y*+1, *x*, *z*−1/2; (xi) *x*, *x*−*y*+1, *z*; (xii) −*x*+*y*+1, −*x*+1, *z*; (xiii) *x*+1, *y*+1, *z*; (xiv) −*x*+1, −*y*+1, *z*−1/2; (xv) *y*, *x*, *z*−1/2; (xvi) *y*, −*x*+*y*+1, *z*+1/2; (xvii) −*y*+1, −*x*+1, *z*.
